# Infant and young child feeding practices in Ethiopia: analysis of socioeconomic disparities based on nationally representative data

**DOI:** 10.1186/s13690-021-00555-x

**Published:** 2021-03-16

**Authors:** Nigatu Regassa Geda, Cindy Xin Feng, Bonnie Janzen, Rein Lepnurm, Carol J. Henry, Susan J. Whiting

**Affiliations:** 1 Center for Population Studies, College of Development Studies, Addis Ababa University, Sidist Kilo Campus, PO Box 1176, Addis Ababa, Ethiopia; 2 School of Public Health, Health Science E-wing, University of Saskatchewan, 104 Clinic Place, Saskatoon, SK S7N 2Z4 Canada; 3 Department of Community Health and Epidemiology, Faculty of Medicine, Dalhousie University, Halifax, Canada; 4 Department of Community Health & Epidemiology, Collège of Medicine, University of Saskatchewan, Saskatoon, Canada; 5 College of Pharmacy and Nutrition, Health Sciences A-wing, University of Saskatchewan, 107 Wiggins Road, Saskatoon, SK S7N 5E5 Canada

**Keywords:** Complementary feeding, Diet diversity, Ethiopia, Infant and young children feeding

## Abstract

**Background:**

Undernutrition among children is a priority area of public health concern in Ethiopia. The purpose of this study was to examine disparities in Infant and Young Child Feeding (IYCF) practices among children 6–23 months.

**Method:**

Data were drawn from the 2016 Ethiopian Demographic and Health Surveys (EDHS). A total of 3240 children aged 6–23 months were used for the present analysis. The outcome variable was IYCF practice score (ranging 0–7) which was constructed based on the linear and combined effects of four sets of variables: breastfeeding, avoidance of bottle feeding, diet diversity score and minimum feeding frequency. IYCF practice score was further recoded into three categories. Proportional odds regression was used to assess the determinants of IYCF category.

**Results:**

The proportional odds regression analysis showed that IYCF scores significantly decreased by 5% (Adjusted Odds Ratio (AOR) = 0.95; 95% CI: 0.93–0.97) for every unit increase in the child’s age. Households with fathers of primary and secondary and above level education were 1.37 times (95% CI: 1.14–1.66) and 1.67 times (95% CI: 1.26–2.23) more likely to be in the high IYCF category than in the poor IYCF category. The likelihood of being in the high IYCF practice category decreased for non-working mothers by 30% (AOR = 0.70; 95% CI: 0.59–0.83) compared to those working in gainful employment. The chance of being in the high IYCF practice category decreased by 29% for households with no access to radio (AOR = 0.71; 95% CI: 0.59–0.85). Those with medium and rich/richer wealth category were 1.54 times (95% CI: 1.22–1.94) and 1.40 times (95% CI: 1.11–1.75) more likely to belong to high IYCF practice category than being in poor IYCF category. For every unit increase in health service utilization, the chance of falling in higher IYCF category increases by 1.15 times (95% CI: 1.08–1.23). The chance of falling in higher IYCF practice category decreases for rural residents by 37% (AOR = 0.63; 95% CI: 0.47–0.84) compared to those residing in urban areas.

**Conclusion:**

For a child, the first two years is the time span during which linear faltering of growth is most prevalent and the period when the process of becoming stunted is almost complete. This study recommends improving access to women for gainful employment, provision of economic support to poor rural women, education and promotion of nutrition messages using most accessible media and boosting the positive role of fathers in child feeding practices.

## Background

Worldwide, undernutrition, such as stunting, wasting and micronutrient deficiencies, is responsible for the death of 3.1 million children under-5 annually [1]. About 28% of children under-5 are stunted in low- and middle-income countries [2], and sub-Saharan Africa is experiencing the highest prevalence (40%) of stunting [2]. The risk of undernutrition increases as the age of the child increases, especially during 3 to 24 months of age [3]. Evidence suggests that suboptimal child feeding practices are the main culprit and one of the leading causes of child undernutrition during this period [2].

The World Health Organization(WHO) recommended exclusively breastfeeding up to 6 months after birth, along with continued breastfeeding up to 2 years or beyond [4]. For proper growth of children, a set of WHO validated core indicators of good Infant Young Child Feeding(IYCF) practices are recommended during the first 24 months of life [4, 5]. The core indicators include breastfeeding, avoidance of bottle feeding, Diet Diversity Score (DDS), and Minimum Feeding Frequency (MFF). Appropriate complementary feeding of children has been promoted as one of the strategies to combat growth faltering and associated ill-health consequences in young children [1, 6]. Despite increased efforts, many low- and middle-income countries do not adhere to this [2, 6]. For instance, only one in three or one in six 6–23-months-old children in sub-Saharan Africa were fed adequately diverse or overall acceptable diets, respectively [7]. Generally suboptimal feedings are practiced in countries having highest burden of malnutrition [8, 9].

In Ethiopia, efforts are being made in implementing a multi-sectoral plan of nutrition intervention (as prescribed in *Sekota* Declaration and National Nutrition Program) to end the high burden of undernutrition in Ethiopia by 2030 [10]. However, the country is still experiencing one of the worst scenarios in IYCF practices. The most recent national survey indicates that nearly half of all infants < 6 months of age were not exclusively breastfed. More than one in four infants still received pre-lacteal feeds that may predispose the child for infectious diseases and risk of diarrhea [11]. In addition, one in two children was not put to breast within one hour after birth. Only 42% of children receive the minimum number of meals, less than 10% of children < 24 months achieve minimum dietary diversity (i.e consumption of at least 4 food groups), and only 6% meet the criteria for the minimum acceptable diet [11]. Studies conducted in different parts of the country also confirmed that the majority of children in the 6–23 month age group were improperly fed (i.e., not exclusively breastfed used pre-lacteal food, were bottle fed, and had inadequate intake of micronutrients) [12, 13]. The very poor IYCF practices explain parts of the reasons for unacceptably higher levels of anthropometric and micronutrient deficiencies among children in Ethiopia [14, 15] in the year 2016 which were, for underweight, stunting and anemia among children under 5 years, 25.0, 38 and 57%, respectively [11].

Studies conducted on this subject in Ethiopia attempted to examine the association between socioeconomic factors and infant and child feeding practices. However, these studies had some limitations worth addressing [12, 16–18]. First, most of these studies were based on a single child feeding indicator, usually diet diversity score [12, 16–18]. This approach reduces the possibility of obtaining a complete picture of IYCF practices in Ethiopia. Second, almost all studies conducted were based on data collected at district or sub-district levels, limiting their generalizability to a larger population. Third, although the application of this outcome measure is not new, its application in a national context (Ethiopia) using robust statistical tools would increase the proper identification of risk factors to enhance our understanding of under and malnutrition of children in Ethiopia.

The present study aims to identify the key risk factors of poor IYCF practices using a comprehensive outcome measure among children age 6–23 months in Ethiopia based on nationally representative data. It is hypothesized that IYCF practice is a function of household attributes.

## Methods and materials

### The study context

Ethiopia has federal system with nine autonomous Regional States, each divided into zones, districts and sub districts/ kebeles [19]. Ethiopia is a predominantly rural country, with almost 80% of its population residing in rural areas [20]. Fueled by a high level of the fertility rate, the country is amongst the fastest growing non-oil economies in the world [20]. In 2016, average life expectancy in Ethiopia was 64 years and under-5 mortality was 62 per 1000 births. Between 1990 and 2017, Ethiopia’s life expectancy at birth increased by 18.8 years [21]. Adult literacy is one of the lowest in Sub Saharan Africa, which is 39% [22]. In 2015, 57% of the population were using improved drinking water sources, and in the same year, sanitation coverage was 28% [23].

### Data sources

The Ethiopian Demographic and Health Survey (EDHS) conducted in 2016 is series of cross-sectional surveys conducted every five years since the year 2000. The survey employed a two-stage stratified cluster sampling. The EDHS collected data from all the nine regions and included a total of 645 enumeration areas (EAs) i.e. an EA is a geographic area consisting of 200–300 households [11]. For the current work, the children’s’ file, which contained entries for 3240 mothers was used. Information about child feeding practices was collected from all women who had at least one child living with them and who was born in the two-years preceding the survey. If there were two eligible children, the younger child was used for this analysis. The EDHS followed previously approved standard protocols, data collection tools and procedures, and participation in the survey was voluntary [24]. More detailed descriptions of the sampling, data collection procedures and detailed set of questionnaires are found at https://dhsprogram.com/publications/publication-fr328-dhs-final-reports.cfm.

Permission to use the data for the purposes of the present study was granted by ORC Macro International (U.S.) and Central Statistics Authority (Ethiopia). Ethical approval was also received by the University of Saskatchewan Behavioral Research Ethics Board.

### Measure of the outcome and exposure variables

The outcome variable is the Infant and Young Child feeding (IYCF) score. It was constructed based on four key indicators: breastfeeding, avoidance of bottle feeding, Diet Diversity Score (DDS), and Minimum Feeding Frequency (MFF). Each of these indicators was computed separately for children of 6–8 months, 9–11 months and 12–23 months, based on the recommendations of WHO and previous studies [5, 25]. Mothers were asked if they were breastfeeding their child during the survey period, if they use bottle feeding, the number of times they feed their index child, and food groups consumed.

For breastfeeding, a score of + 2 was given to children 6–8 and 9–11 months, and + 1 was given for children aged 12–23-month-old infants who were breastfed. A score of 0 was given to non-breastfeeding infants at all ages. Diet diversity score was measured based on the consumption of the seven food groups over the last 24 h (0 = no, yes = 1) according to the WHO’s IYCF guidelines [5, 26]. The food groups are: (i) grains, roots, and tubers; (ii) legumes and nuts; (iii) vitamin A rich fruits and vegetables; (iv) flesh foods (meat, fish, poultry and liver/organ meats); (v) eggs; (vi) dairy products (milk, yogurt, cheese); (vii) other fruits and vegetables [5, 26]. The DDS value was obtained by summing up the dietary diversity score, which ranges from zero to seven, where a higher score indicates better diet diversity.

Feeding frequency was defined as receiving solid, semi-solid or soft foods for a minimum number of times in the previous day. The scoring of the feeding frequency varied by child age and breastfeeding status [26, 27]. For 6–8-month-old infants, feeding frequency was used to account the times for complementary foods, excluding breastfeeding and formula-feeding. Scores of + 1 and + 2 were given for infants who met the lower end of the recommendation and those who meet or exceed the higher end, respectively [27]. For infants 6–8 months, those with 0–1-time per day complementary food consumption was given a score of 0, 2 times a day received a score of + 1 and 3 times or more per day received a score of + 2. Similar procedures were used for children 9–11 months. The index was calculated by adding up the scores. For each age category, the combined score ranged between 0 and 6.

Avoidance of bottle feeding was included as an indicator in this study as desirable feeding practices. Bottle feeding is usually considered as unfavorable practice as improper sanitation and formula preparation with bottle feeding can introduce microorganisms to the infant that increase the child’s risk of illness and malnutrition [28]. This indicator takes a value of + 1 if mothers did not use bottle feeding and ‘0’ otherwise.

The index was divided into three categories based on median values: a score of 0–2 was considered low, 3–4 as medium and 4+ as high. Nearly identical procedures of categorization was used in other similar studies [25]. The variables and scoring system used are shown in Table 1. Higher categories indicate more favorable feeding practices.

**Table 1:** Variables and scoring system used to construct the IYCF Score

Variable	IYCF Scoring		
	6–8 months	9–11 months	12–23 months
Breastfeeding	Yes = 2	Yes = 2	Yes = 1
	No = 0	No = 0	No = 0
Bottle feeding	Yes = 0	Yes = 0	Yes = 0
	No = 1	No = 1	No = 1
Diet diversity	0–1 = 0	0–2 = 0	0–2 = 0
	2 = 1	3 = 1	3 = 1
	3 + = 2	4 + =2	4 + =2
Feeding frequency	0–1 = 0	0–2 = 0	0–2 = 0
	2 = 1	3 = 1	3 = 1
	3 + =2	4 + =2	4 = 2
			5 + =3
**Total score**	**7**	**7**	**7**

The choice of potential factors associated with IYCF practices was guided by the literature review and available information on variables collected by EDHS questionnaires. The exposure variables were categorized into three major groups: individual characteristics (age of the child, sex of the child, parental education, work status, service utilization scores), household characteristics (household wealth status, access to radio, religion, type of family structure, presence of other children under-5, children ever born) and a community variable (place of residence). Most of these exposure variables were used as originally coded in EDHS, while some of them were recoded to fit the present analysis. Household wealth was used as a proxy to household income and was estimated in the EDHS with ownership of household assets [24]. Service utilization score was constructed from the affirmative responses of six key child health interventions associated with the most recent birth. It included Antenatal Care (ANC) service (> 4 visits), delivery of the last child at health facilities, postnatal care services, vitamin A intake, iron supplementation and intake of deworming by the index child. This variable was a count ranging from 0 to 6 where zero represents none of the services and 6 stands for all services were used.

### Statistical analysis

The distribution of various sociodemographic variables was described using frequencies and proportions. Due to the categorical nature of the outcome variable, the analysis started by checking the proportional odds regression assumption using a score test [29] to determine if the proportional odds model is appropriate in this analysis. As the *p*-value of the score test is higher than 5%, the proportional odds assumption is not violated, so the proportional odds regression model is appropriate in the present study. Correlations among the explanatory variables was checked using the Variance Inflation Factor (VIF) [29]. The bivariate proportional odds regression was conducted to select the most promising explanatory variables for multivariable proportional odds regression. Variables with a p-value< 0.20 in the bivariate analysis were selected for entering the initial multivariable proportional odds. Akaike Information Criteria (AIC) was used to select the final model. Two-way interactions were assessed for some significant variables. Odds ratios with 95% confidence intervals were calculated for each factor in the ordered logistic regression model.

All analyses were weighted using the weight variable computed by the Central Statistics Authority of Ethiopia [11]. Analysis of the data were carried out using STATA version 13 [30].

## Results

Table 2 displays study participant characteristics (*n* = 3240). About 88% of the respondents were residing in rural areas. The proportion of female children was slightly higher (52%). Just over 60% of mothers (61%) had no education, 30.3 and 8.5% reported primary and post-secondary education, respectively. Among fathers, 38.9 and 13.4% had a primary and secondary level of education, respectively. In 60% of the households, respondents reported the presence of other under-5 children. Only about a quarter of the mothers were working outside their home during the survey period. Close to 72% of the households did not have access to media (radio). Most of the respondents had a Muslim religious background (41%) followed by Orthodox Christians (35%). Half of the respondents reported having 0–3 children ever born. The mean age of the mothers was 28.63(SD = 6.59). A greater proportion (44.2%) of the households were in the poorest/poorer households compared to those living in richest/richer households (34.1%) (see Table 2).

**Table 2:** Results of bivariate proportional odds regression for predictors of Infant and Young Child Feeding practices, Ethiopia, *n* = 3240

			95% C.I. for OR		
Characteristics	N (%)	OR	Lower	Upper	***p***-values
**Sex of the child**					
Male ^RC^	1556 (48.0)				
Female	1684 (52.0)	0.920	0.760	1.115	0.397
**Age of the child in months**	14 (8) ^a^	0.917	0.907	0.927	0.000
**Education of mother**					
No education ^RC^	1981 (61.1)				
Primary level	983 (30.3)	1.798	1.435	2.253	0.000
Secondary and above	276 (8.5)	4.241	3.290	5.465	0.000
**Education of father**					
No education ^RC^	1547 (47.7)				
Primary	1260 (38.9)	1.911	1.510	2.417	0.000
Secondary and higher	433 (13.4)	3.698	2.900	4.716	0.000
**Presence of other under-5 children**					
No ^RC^	1301 (40.1)				
Yes	1940 (59.9)	0.670	0.553	0.813	0.000
**Mother’s Work status**					
Working ^RC^	870 (26.8)				
Not working	2371 (73.2)	0.539	0.441	0.659	0.000
**Children ever born**					
0–3 ^RC^	1621 (50.0)				
4–6	997 (30.8)	0.683	0.547	0.854	0.001
7 and above	622 (19.2)	0.560	0.418	0.751	0.000
**Access to radio**					
Yes ^RC^	919 (28.3)				
No	2322 (71.7)	0.386	0.316	0.472	0.000
**Religion**					
Orthodox ^RC^	1129 (34.8)				
Muslim	1315 (40.6)	0.740	0.597	0.917	0.006
Others	797 (24.6)	0.688	0.522	0.908	0.008
**Type of family structure**					
Monogamy ^RC^	2756 (85.1)				
Polygamy	319 (9.8)	0.821	0.604	1.117	0.209
**Health service utilization score**	1 (3) ^a^	1.369	1.281	1.463	0.000
**Wealth index**					
Poor/ poorer ^RC^	1432 (44.2)				
Medium	705 (21.7)	2.206	1.637	2.972	0.000
Rich/ richer	1104 (34.1)	3.854	3.086	4.814	0.000
**Place of residence**					
Urban ^RC^	395 (12.2)				
Rural	2845 (87.8)	0.288	0.235	0.354	0.000

IYCF index values before categorization: mean = 2.36; SD = 1.19; median = 2; minimum = 0; maximum = 6; IYCF score after categorization: Poor: 783 (24.2%); medium: 1967(60.7%); high: 490(15.1%)

^a^Median and IQR values; RC = Reference category

**Fig. 1:**
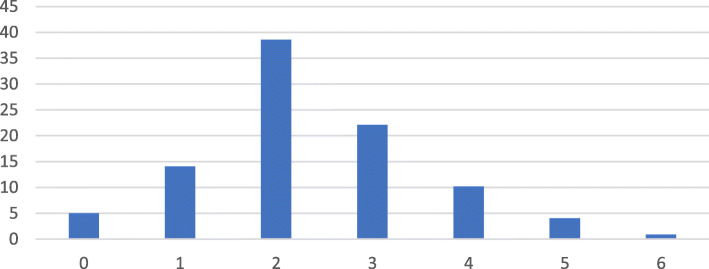
Distribution of IYCF scores

Figure 1 further shows that about 85% of the children aged 6–23 months in Ethiopia had poor IYCF (i.e., score less than 4 out of 7 possible scores).

Table 3 presents the results of ordered logistic regression for key determinants of IYCF. Multicollinearity analysis among the explanatory variables indicates that none of them had a significant relationship. The scatterplot between the dependent and continuous independent variables confirmed linearity assumptions are met.

**Table 3:** Results of multivariable proportional odds regression for predictors of Infant and Young Child Feeding practices, Ethiopia

		95% C.I. for AOR		
Characteristics	AOR	Lower	Upper	*p*-values
**Age of the child (in months)**	0.953	0.935	0.972	0.000
**Education of mother**				
No education ^RC^				
Primary level	1.112	0.908	1.360	0.304
Secondary and above	1.347	0.992	1.829	0.056
**Education of father**				
No education ^RC^				
Primary	1.373	1.139	1.655	0.001
Secondary and higher	1.673	1.256	2.229	0.000
**Presence of other under-5 children**				
No ^RC^				
Yes	0.892	0.756	1.054	0.180
**Mother’s Work status**				
Working ^RC^				
Not working	0.699	0.589	0.831	0.000
**Number of children ever born**				
0–3 ^RC^				
4–6	0.997	0.826	1.204	0.976
7 and above	0.977	0.773	1.234	0.847
**Health service utilization score**	1.154	1.084	1.229	0.000
**Access to radio**				
Yes ^RC^				
No	0.709	0.588	0.854	0.000
**Religion**				
Orthodox ^RC^				
Muslim	1.136	0.907	1.423	0.267
Others	0.785	0.626	0.985	0.036
**Wealth index**				
Poor/ poorer ^RC^				
Medium	1.540	1.222	1.940	0.000
Rich/ richer	1.396	1.111	1.752	0.004
**Place of residence**				
Urban ^RC^				
Rural	0.630	0.474	0.837	0.001
**Cons**	0.429	0.256	0.719	0.001

*RC* Reference category

The significant individual-level variables were age of the child, maternal and paternal education, maternal work status and maternal health service utilization. The IYCF score significantly decreases by 5% (AOR = 0.953; 95% CI: 0.935–0.972) for every unit increase in the child’s age. Children with fathers of primary and secondary and above education were 1.37 times (95% CI: 1.139–1.655) and 1.67 times (95% CI: 1.256–2.229) more likely to belong in higher IYCF score category than being in the poor category. The likelihood of falling in higher IYCF practice decreases for children from non-working mothers by 30% (AOR = 0.699; 95% CI: 0.589–0.831) compared to those working in gainful employment.

Among the household variables, access to radio, religion and household wealth were significantly associated with IYCF practices. The chance of falling in higher IYCF practice decreases by 29% for households with no access to the radio (AOR = 0.709; 95% CI: 0.588–0.854). Children living with parents of ‘other religion’ followers were 21% less likely to belong to higher feeding categories (AOR = 0.785; 95% CI:0.626–0.985) compared to those from parents of Orthodox Christian followers. Those from medium and rich/richer wealth households were 1.54 times (95% CI: 1.222–1.940) and 1.40 times (95% CI: 1.111–1.752) more likely to belong to better IYCF practice category than being in poor IYCF category, respectively.

The two community and service-related variables (residence and health service utilization scores) have become significantly associated with IYCF practice. For every unit increase in health service utilization, the chance of a child falling in a higher IYCF category increases by 1.15 times (95% CI: 1.084–1.229). The chance of falling in higher IYCF practice category decreases for rural children by 37% (AOR = 0.630;95% CI: 0.474–0.837) compared to those residing in urban areas. Finally, all possible two way interaction effects of selected variables were tested, but none of them showed promising results.

## Discussion

This study has primarily aimed at assessing the factors associated with IYCF practices based on the most recent nationally representative data of Ethiopia. The study used a comprehensive measure of IYCF practices among children 6–23 months. The results of our study provide several noteworthy findings regarding the predictors of infant and young children’s feeding practices in Ethiopia. The regression analysis revealed a range of individual, household, and community variables affecting IYCF. Age of the child, paternal education, maternal work status, children ever born by mothers and health service utilization were the individual-level variables strongly associated with IYCF practices. Among the household-level variables, household wealth, access to radio and religion significantly predicted IYCF practices. Place of residence was important service-related and community-level predictors of IYCF practices in Ethiopia.

About 85% of the children aged 6–23 months in Ethiopia were found to have a very poor or poorer IYCF score (i.e., score less than 4 out of 7 possible scores). There are no comparable national-level studies conducted in Ethiopia using the same outcome measure; however, the finding is consistent with local studies in Ethiopia which reported unacceptably low prevalence for nearly all indicators. For instance, a study conducted in Southern Ethiopia documented that only 50.9% of mothers introduced timely complementary feeding at 6 months, 22.2% achieved minimum dietary diversity, only 12% of them received the minimum acceptable diet; 39.8% of mothers fed their children using bottle [13]. The figure in this study is much lower than reported in other studies in Africa and elsewhere in the world. For example, in Zambia, the proportion of children aged 6–23 months given more than four food groups in a day was 37.1% [8], 29.5% in Uganda and 16% in India [9].

An inverse association was found between the age of the child and IYCF practice i.e., the likelihood of a child falling in higher IYCF practice decreases with the age of the child. One possible reason for the inverse association could be reduced attention as the child grows, possible poor intrahousehold food distribution, early cessation of breastfeeding and resource limitations. Accordingly, stunting is less common in early infancy as most children are being breastfed [31, 32]. The risk of impaired growth increases as breastfeeding is discontinued without adequate complementary feeding and with poor diet diversity [31]. However, it is important to note that studies around the world have reached inconsistent conclusions. For instance, a reverse trend was observed in Sierra Leone and Uganda [33]. Other studies also reached a similar conclusion [31, 32], while a few others reported a positive relationship between the age of children and IYCF practice [16]. Such inconsistent findings could arise from differences in data collection periods as affected by seasonality of production and consumption.

The findings indicated that paternal education had a stronger and significant association with IYCF practices. This is not surprising as some studies around the world have reported that educated fathers are more involved with issues of diet/ nutrition and parenting behaviors, which contribute to the overall health and well-being of their young children [34, 35]. Additionally, educated fathers provide a higher household income, more freedom and supports, higher social status and stability, and more opportunities for their wives and children [36, 37]. Allen and Daly [37] reported that when mothers are supportive of their spouse’s parenting (provide encouragement, expect and believe parenting is a joint venture), fathers would be more likely to be involved with, and responsible for their children [37]. Fathers’ education could also result in greater maternal autonomy [38] which in turn positively affects gender roles and good intra-household food distribution, impacting the nutrition of both women and young children [39].

In the present study, a reverse association was found that non-working mothers were less likely to fall in higher IYCF practice categories than working mothers. Previous national level studies conducted around the world reached inconsistent conclusions. In a study of IYCF practices in Sub Saharan African countries, six of the ten countries demonstrated positive odds ratios (AOR = 1.04–1.29) in the relationship between economic empowerment(work status was a component) and the probability of meeting the minimum feeding frequency criterion [33]. A study in the Philippines found that maternal contribution to household income and her control over such income were significantly associated with increased weekly household food expenditure after controlling for potential confounders [40]. Women’s employment passes through different pathways to influence good feeding practices. The most prominent pathways could be the fact that working outside home provide women their freedom of mobility and decision making regarding interpersonal or family affairs [33]. This will, in turn, enhances the ability of mothers to acquire resources, such as information and support from friends and relatives [41]. Higher economic empowerment of women through employment is expected to be associated with increased financial access to foods and increased food distribution to children [40]. Contrary to this finding, a national level study conducted in Madagascar reported that infants whose mothers did not work outside the home were more likely to have better food consumption practices [42]. The Madagascar study and other scholars argue that working mothers may have less time available for child care and feeding [43–45]. The benefit of increased income and control over income and the cost of reduced time is often recognized as a trade-off between maternal employment and child care [33]. Employment of mothers may reduce the time for breastfeeding of younger infants, but this could be advantageous to older children since they will have access to better nutritional status compared with their counterparts of unemployed or less financially autonomous mothers [33, 46].

Access to media, either printed or non-printed, is usually mentioned in previous studies as a key determinant of behavioral change which positively influences household nutrition and food consumptions [9, 47]. Given that two-thirds of the respondents had no education, the present study solely focused on the effects of exposure to radio on IYCF practices in Ethiopia. Interestingly, we found that households with no access to radio had a significantly lower chance of practicing good IYCF compared to those having some exposure to radio. The finding is comparable with a nationwide study in India [9] which reported that mothers who were less exposed to media had less knowledge of nutrition. In another study, infants born to mothers who do not read newspaper at all were less likely to obtain iron-rich foods [42]. In a study conducted in Southern Ethiopia, children were found to have higher dietary diversity score in situations where mothers had received IYCF information on mass media in the last month [47].

The findings on the strongly significant reverse association between household wealth and IYCF practices is consistent with previous studies conducted in different parts of the world. Cross country studies confirmed that increased family wealth was associated with better chances of meeting minimum dietary diversity [48, 49]. Higher household wealth generally means higher purchasing power and having access to more diverse food and more resources to be allocated to childcare and nutrition [42].

The effects of religion on IYCF practice is another noteworthy finding in this study. Ethiopia is a home of diverse cultures and religions which dictates different food habits and practices [16], and the impacts of religion on IYCF could vary across different settings. A recent study conducted in three countries (Uganda, South Africa, and Burkina Faso) showed that a strong relationship between infant feeding practice and religious beliefs [50]. One of the most plausible pathways for religion to influence IYCF practices is through its imposition of health beliefs and food taboos [51]. From a study conducted in Tajikistan, it was reported that religion and culture dictated food taboos restricted consumption of key staple foods and nutrient-rich fruits and vegetables for members of Tajik household [51].

Another remarkable finding of the present study is the significant positive association between health service utilization and IYCF practice scores. This is consistent with previous studies conducted in Ethiopia which reported that health service utilization by households had a significant positive association with the timely initiation of complementary feeding [17, 18]. A study based on national data of Nepal indicated that child feeding was higher among women who had four or more ANC visits and who delivered their child in the health facilities [52]. Another Nepalese study confirmed that antenatal visits, institutional delivery, and postnatal visits, have significantly positive impacts on child feeding practices, especially initiation of breastfeeding and complementary feeding for children in 6–8 months. It is well documented that adequate antenatal visits during pregnancy provide an exceptional opportunity for mothers to have counselling on breastfeeding practices [52, 53]. The findings suggest that increased promotion of childbirth at health facility and education about child feeding, at facility level is one of the most promising and low-cost strategies to improve the overall IYCF practices in low income countries like Ethiopia.

Finally, the present study found that IYCF was significantly affected by the type of residence. Those residing in rural areas had a lower chance of falling in higher IYCF practice categories compared to those living in urban areas. Consistent with this finding Haina and Colleagues [42] reported that children living in rural areas had higher odds of having inadequate consumption of iron-rich food [42].

### Strengths and limitations

Given the very high prevalence of stunting and micronutrient deficiencies among Ethiopian children, which in most part occurred due to poor IYCF practices, the findings could prove useful on a national scale for the planning, targeting, monitoring and evaluating of future nutrition sensitive and specific programs. As this study is the first of its kind in Ethiopia using a comprehensive IYCF outcome variable, its contribution to literature in the subject and program implementation is great. The study also has some methodological limitations worth mentioning. First, the EDHS survey employed a cross-sectional design, where data on the exposure and outcomes were collected at the same point in time. This limits assessment of cause-effect relationship between the explanatory and outcome variables. Second, there are possibilities of omission, under-reporting, or improper reporting of important information as most of the respondents had no education. Finally, the outcome variable for the present study was a score variable constructed based on specific WHO recommended indicators. The construction of this index may alter the average values of the original individual indicators.

## Conclusion

The study indicated that the prevalence of both individual indicators and the combined IYCF practices are unacceptable low among a significant proportion of households in Ethiopia. The study further reiterated that several individual, household and community level characteristics are associated with inappropriate feeding practices in Ethiopia. Given that the first two years is the time span during which faltering growth is most prevalent and the period when the process of becoming stunted almost completes, it is extremely important for health professional to pay more attention to this critical period and implement both sensitive and specific nutrition interventions. The finding implies that significant improvement in IYCF can be made through improving access to women for gainful employment, provision of economic support to poor rural women/households, education and promotion of nutrition messages at health facilities or using most accessible media, and boosting the positive role of fathers in child feeding practices.

## Data Availability

The datasets used for this study are made available from ICF international/DHS program at https://dhsprogram.com/data/Access-Instructions.cfm. Thus, administrative permissions were required to access the raw data from this organization. Public access to the database is open upon permission.

## Abbreviations

ANC: Antenatal Care
CSA: Central Statistics Authority
DDS: Diet Diversity Score
DHS: Demographic and Health Surveys (DHS).
EDHS: Ethiopian Demographic and Health Surveys
IYCF: Infant and Young Child Feeding
MFF: Minimum Feeding Frequency
WHO: World Health Organization
